# Prognostic Nomogram for the Overall Survival of Patients with Newly Diagnosed Multiple Myeloma

**DOI:** 10.1155/2019/5652935

**Published:** 2019-04-08

**Authors:** Yue Zhang, Xiao-Lei Chen, Wen-Ming Chen, He-Bing Zhou

**Affiliations:** ^1^Department of Hematology, Beijing Chaoyang Hospital, Capital Medical University, Beijing, China; ^2^Department of Hematology, Beijing Luhe Hospital, Capital Medical University, Beijing, China

## Abstract

To establish a nomogram for predicting the overall survival (OS) of patients with newly diagnosed multiple myeloma (MM), 304 patients with newly diagnosed MM were recruited between June 1, 2010, and June 30, 2015, from the Beijing Chaoyang Hospital, Capital Medical University, and randomly divided into training (n=214) and validation (n=90) cohorts. The Kaplan-Meier method and the Cox proportional hazards regression model were used to evaluate the prognostic effects of multiple clinical and laboratory parameters on survival. Significant prognostic factors were combined to build a nomogram. The discriminative ability and predictive accuracy of the nomogram were evaluated using the index of concordance (C-index) and calibration curves and compared with the five staging systems currently used for MM. Multivariate analysis of the training cohort revealed that the age at diagnosis, clonal bone marrow plasma cells, serum lactate dehydrogenase, serum *β*2-microglobulin, and del (17p) were independent risk factors for OS and were used to establish the nomogram. The C-index value of the nomogram for predicting OS was 0.749, which was significantly higher than the C-indices of the five most common staging systems currently used for MM. In the validation cohort, the C-index for nomogram-based predictions was 0.711 for OS, and the nomogram discrimination was better than the above mentioned five staging systems (*P*<0.001). All calibration curves revealed good consistency between predicted and actual survivals. The proposed nomogram is more accurate in predicting the prognoses of patients with newly diagnosed MM.

## 1. Introduction

Multiple myeloma (MM) is a clonal plasma cell disease, with the hallmark features of accumulation and proliferation of malignant plasma cells in the bone marrow (BM). The spectrum of symptoms arises from both the tumor load in the BM and the excessive production of immunoglobulins. Typical symptoms of MM include hypercalcemia, renal insufficiency, anemia, and bone lesions [1] and are known by the moniker “CRAB” [2]. The occurrence of MM is multistep and multistage, and the gradual accumulation of genetic abnormalities leads to aggressive tumor growth; the clinical prognosis of MM is highly heterogeneous [3]. Presently, the patients with high-risk MM are treated by proteasome inhibitors, immunomodulator drug-based chemotherapy, combined with autologous stem cell transplantation (ASCT) per the high-risk MM model, but patients with relapsed or refractory MM may still experience rapid progress. However, for patients with low-risk MM, the use of a new unified drug combination treatment allows these patients to obtain certain survival advantages, but has resulted in significant treatment-related adverse reactions. Therefore, an accurate prognostic model for predicting survival is needed to guide the treatment selection for patients with newly diagnosed MM.

Presently, the staging systems of MM mainly include the Durie-Salmon staging system (D-S) [4], the International Staging System (ISS) [5], the Revised-International Staging System (R-ISS) [6], the International Myeloma Working Group (IMWG) risk stratification [7], and the Mayo Stratification of Myeloma and Risk-Adapted Therapy (mSMART) [8]. Historically, the D-S system was the first staging system for MM, reflecting the tumor burden and the clinical course of MM. The ISS staging system, based on two simple laboratory tests, is mainly used for the evaluation of the prognoses of patients; however, the evaluation needs further improvements in the era of new drugs. Both these staging systems do not assess cytogenetic markers and do not have the ability to evaluate prognosis in the early stages of disease. The R-ISS staging system is newly revised for prognostic evaluation, wherein cytogenetics and lactate dehydrogenase (LDH) are prognostic factors that are independent of the ISS staging system. The ISS staging system did not incorporate chromosomal abnormalities which is one of the most important prognostic factors in MM. In patients with newly diagnosed MM, high-risk disease is characterized by the presence of del (17p), t (4;14), or t (14;16) detected by interphase fluorescence in situ hybridization (iFISH). High serum LDH has been linked to shorter overall survival (OS) in MM and likely reflects disease aggressiveness and drug resistance and may also be an indicator of extramedullary disease. The R-ISS has also significantly better discriminative power than the ISS in MM patients treated with novel agents as a primary therapy [9]. The IMWG and mSMART systems have been widely used; the mSMART system was first proposed in 2007 by the Mayo clinic with cytogenetic analysis as the foundation and was updated in 2013. In 2014, the IMWG consensus was applied to stratify patients according to risk, using ISS and iFISH. However, it remains uncertain whether these staging systems are suitable for Chinese patients with MM. Additionally, all these staging systems are not suitable for every single patient with different prognostic factors.

The nomogram is a graphical representation of a mathematical model, wherein information on several characteristics is combined to predict a specific endpoint. The convenient graphical representation of a nomogram allows predictions to be obtained easily and quickly in practice [10]. By integrating various important factors, a nomogram can provide individualized estimates of the probability of an event, such as the individual probabilities of disease recurrence or deaths in patients [11]. Therefore, the nomogram has become a reliable tool for predicting the clinical outcomes of many types of cancers [12–15].

However, published literature does not currently include nomograms to predict the survival outcomes in patients with MM. Our study is the first attempt to establish a prognostic nomogram for patients with newly diagnosed MM, based on clinical and laboratory data, to determine whether the nomogram can predict survivals more accurately, when compared with the currently used staging systems.

## 2. Materials and Methods

### 2.1. Patients and Study Design

A cohort of 304 patients with newly diagnosed MM was recruited from the Beijing Chaoyang Hospital, Capital Medical University between June 1, 2010, and June 30, 2015. All patients were diagnosed according to the IMWG diagnostic criteria [16]. All patients were treated with at least one novel agent, followed by ASCT if eligible. The following information was obtained for each patient: age at diagnosis, sex, clonal BM plasma cells, hemoglobin, serum albumin, serum LDH, serum creatinine, serum calcium, serum *β*2-microglobulin, iFISH analysis of 1q21 gain, del (17p), t (4;14), t (11;14), t (14;16), and survival information. Patients for whom data on any of these characteristics could not be obtained were excluded. All patients were randomized to two groups. Training cohort and validation cohort were randomly assigned in a 7:3 ratio (training cohort, n=214; validation cohort, n=90).

The study was censored on June 30, 2017. This study was approved by the ethics committee of the Beijing Chaoyang Hospital, and informed consents were obtained from all patients.

### 2.2. iFISH Analysis

Five-mL BM samples were obtained from the patients with MM at the time of diagnosis. Mononuclear cells were condensed by the Ficoll density gradient centrifugation method (Ficoll-Paque PLUS; GE Healthcare Bio-Sciences AB, Uppsala, Sweden). Plasma cells were purified using anti-CD138-coated magnetic beads (Miltenyi technology, Bergisch Gladbach, Germany), enabling a plasma cell purity >90% [17]. These purified plasma cells were analyzed using DNA probes (Vysis / Abbott Molecular, Des Plaines, IL), to detect the following cytogenetic aberrations: del (17p), t (4;14), t (11;14), and t (14;16) [18]. Gains of 1q21 were detected by the LSI 1q21 FISH Probe Kit (China Meditech, Beijing, China), as described previously [19]. A total of 200 interphase nuclei were analyzed. The cutoff value for each iFISH probe was set as >5%.

### 2.3. Follow-Up

Patients were observed every 3 months. OS was defined as the time from diagnosis to death from any cause. In the analysis of OS, patients who were alive at the last follow-up were classified as censored.

### 2.4. Categorization of Patients in the Currently Used Staging Systems

Patients were categorized according to the five current staging systems, including ISS, R-ISS, D-S, mSMART, and IMWG.

### 2.5. Statistical Analysis

The statistical analysis was performed using IBM SPSS statistics 22 software (SPSS Inc., Chicago, IL, USA). The Kaplan-Meier method and the Cox proportional hazards regression model were used to determine survival-related factors. These factors were observed to have significant associations with survival in univariate or multivariate analyses.

A nomogram was developed based on the results of multivariate analysis, using R 3.4.1 software (Institute for Statistics and Mathematics, Vienna, Austria; http://www.r-project.org/). The “rms” R library (cran.r-project.org/web/packages/rms) was used to construct survival models and compare the nomogram to the other staging systems. The nomogram was subjected to 1000 bootstrap resamples for internal validation in the training cohort and external validation in the validation cohort, respectively. Marginal estimates and model average prediction probabilities were used to create calibration curves. In a perfectly calibrated model, the predictions should fall on the diagonal 45° line of the calibration plot. Predictive performance was assessed using the index of concordance (C-index), which resembles the area under the curve (AUC), but appears to be better suited for censored data [20]. A larger C-index indicates more accurate prognostic predictions [21].* P* values were two-sided, and* P *values <0.05 indicated statistical significance.

## 3. Results

### 3.1. Clinical and Laboratorial Characteristics of the Patients

A total of 304 patients with newly diagnosed MM were identified for this study. Patients were randomly divided into a training cohort (n=214) and a validation cohort (n=90). The clinical and laboratory characteristics of patients in the training and validation cohorts are listed in Table 1.

### 3.2. OS in the Training Cohort

The median OS was 33 months (range, 1−84), and the 1-, 2-, and 3-year OS rates were 86.9%, 78.0%, and 67.5%, respectively.

### 3.3. Independent Prognostic Factors in the Training Cohort

In the training cohort, 214 patients were included in univariate and multivariate analyses to determine the predictors of OS. The results of the univariate analysis showed that the age at diagnosis, clonal BM plasma cells, serum albumin, serum LDH, serum *β*2-microglobulin, 1q21 gain, and del (17p) were correlated with OS (*p* <0.05). The Cox proportional hazards regression model was used to further explore the influences of these variables. Multivariate analyses demonstrated that the age at diagnosis, clonal BM plasma cells, serum LDH, serum *β*2-microglobulin, and del (17p) were independent risk factors for OS (Table 2).

### 3.4. Prognostic Nomogram for OS

**Figure 1:**
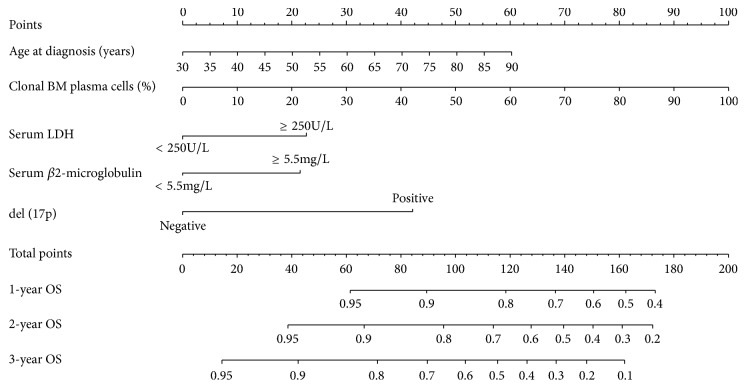
Nomograms for the prediction of the 1-, 2-, and 3-year overall survivals in patients with newly diagnosed multiple myeloma. To use the nomogram, first, the position of each variable on the corresponding axis should be found. Next, a line to the points axis for the number of points should be drawn. Then, the points from all the variables should be added. Finally, a line from the total points axis should be drawn to determine the overall survival probabilities at the lower line of the nomogram. Abbreviations: BM, bone marrow; LDH, lactate dehydrogenase; OS, overall survival.

The prognostic nomogram included all the significant independent factors of the Cox proportional hazards regression model in the training cohort. It established scoring criteria according to the hazard ratio (HR) values of all prognostic factors and gave a score for each level of prognostic factors. Then, the line segments with scale are drawn on the same plane according to a certain proportion and displayed in a graphical way. The prognostic nomogram for 1-, 2-, and 3-year OS is shown in Figure 1. By adding up the scores associated with each variable, and projecting total scores to the bottom scale, probabilities can be estimated for 1-, 2-, and 3-year OS. With the aid of a nomogram, it was possible to effectively predict prognoses according to individual patient characteristics.

### 3.5. Validation of the Nomogram

**Figure 2:**
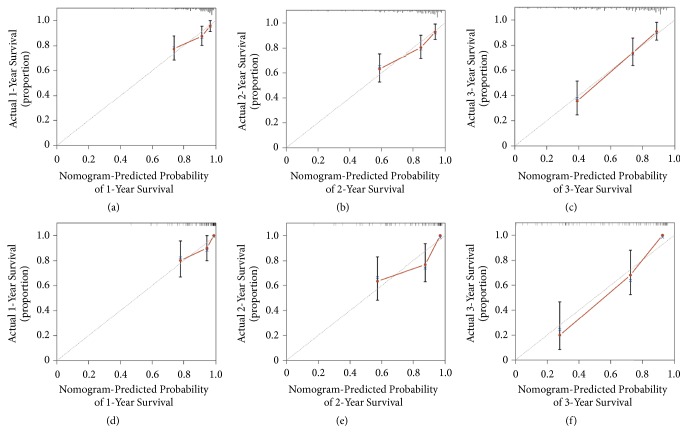
The calibration curves for the predictions of overall survivals in the training ((a)−(c)) and the validation ((d)−(f)) cohorts at 1, 2, and 3 years after diagnosis. The dashed line represents perfect correspondence between the probabilities predicted by the nomogram (x-axis) and calculated by Kaplan-Meier analysis (y-axis), respectively.

Validation of the nomogram was performed using bootstrap analyses with 1000 resamples, processed both internally and externally. Analysis of the internal validation cohort (training cohort) showed a C-index value of 0.749 (95% confidence interval [CI], 0.693−0.805) for nomogram-based predictions of OS. Similarly, in the external validation cohort (validation cohort), the C-index value for predicting OS was 0.711 (95% CI, 0.650−0.772). These findings indicate that the nomogram model was reasonably accurate. The internal and external calibration curves demonstrated good agreement between the predicted and observed values for 1-, 2-, and 3-year OS in both the training and validation cohorts (Figure 2).

### 3.6. Comparison of Predictive Accuracy for OS between the Nomogram and the Different Staging Systems

**Figure 3:**
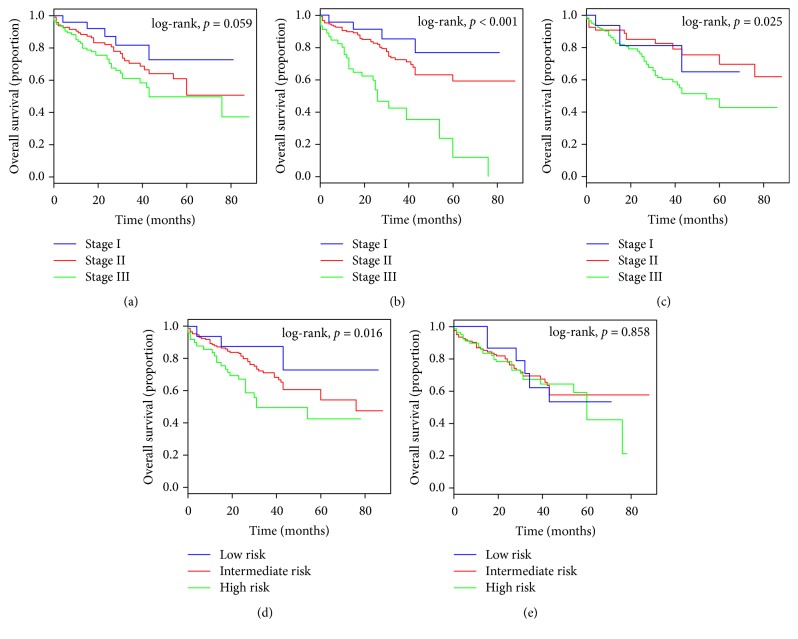
Kaplan-Meier survival curves of the patients with MM in the training cohort, categorized by different staging systems. (a) International Staging System (ISS); (b) Revised International Staging System (R-ISS); (c) Durie-Salmon (D-S); (d) Mayo Stratification of Myeloma and Risk-Adapted Therapy (mSMART); (e) International Myeloma Working Group (IMWG).

As shown in Figure 3, the ISS was unsatisfactory in stratifying patients between stages I, II, and III in the training cohort (Figure 3(a)). The D-S was unsatisfactory in stratifying patients between stages I and II (Figure 3(c)). However, the R-ISS showed good prognostic stratification for the patients in the training cohort between stages I, II, and III (Figure 3(b)). The mSMART showed good prognostic stratification for the patients in the training cohort between low-, intermediate-, and high-risk categories (Figure 3(d)). However, the IMWG was unsatisfactory in stratifying patients between the low-, intermediate-, and high-risk categories (Figure 3(e)).

**Figure 4:**
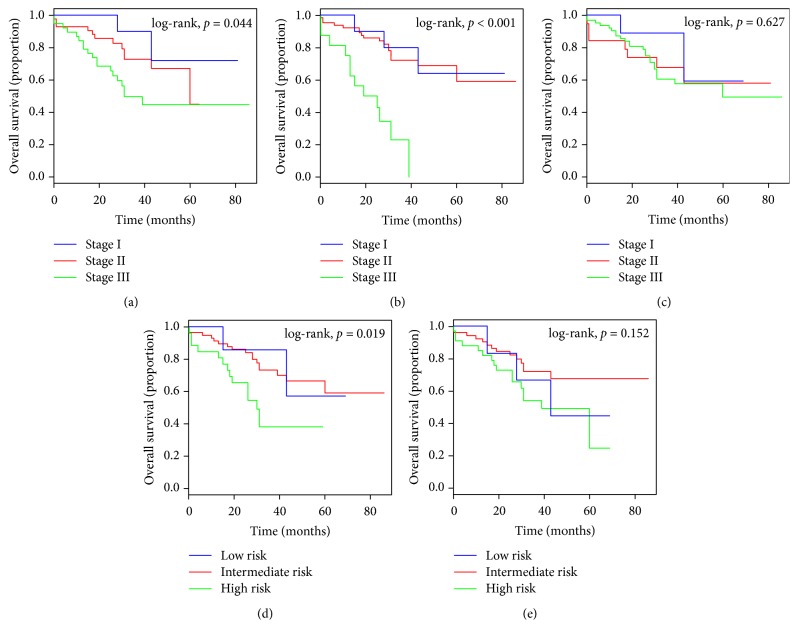
Kaplan-Meier survival curves of the patients with MM in the validation cohort, categorized by different staging systems. (a) International Staging System (ISS); (b) Revised International Staging System (R-ISS); (c) Durie-Salmon (D-S); (d) Mayo Stratification of Myeloma and Risk-Adapted Therapy (mSMART); (e) International Myeloma Working Group (IMWG).

As shown in Figure 4, the ISS and the D-S were also unsatisfactory in stratifying patients between stages I, II, and III in the validation cohort (Figures 4(a) and 4(c)). The R-ISS showed a poor prognosis in stage III and was unsatisfactory in stratifying patients between stages I and II (Figure 4(b)). The mSMART was unsatisfactory in stratifying patients between low- and intermediate-risk categories in the validation cohort (Figure 4(d)), and the IMWG was unsatisfactory in stratifying patients between the low-, intermediate-, and high-risk categories (Figure 4(e)).

When compared with the ISS, R-ISS, D-S, mSMART, and IMWG staging systems, the nomogram displayed higher levels of accuracy in predicting survivals in both the training and the validation cohorts. The C-index of the nomogram in the training cohort was higher than the C-indices of the ISS, R-ISS, D-S, mSMART, and IMWG systems (*P*<0.001). Similarly, in the validation cohort, the C-indices of the ISS, R-ISS, D-S, mSMART, and IMWG were lower than the C-index of the nomogram (*P*<0.001) (Table 3). These results suggest that the nomogram is a more accurate and useful tool for the prediction of OS in patients with MM.

## 4. Discussion

The nomogram is a graphical representation of a mathematical model that combines biological and clinical variables to determine the probabilities of clinical events. The nomogram is widely used for predicting the prognoses in cancer patients, mainly because of its ability to estimate the probability of an event, such as death or recurrence, that is tailored to the profile of an individual patient. User-friendly graphical interface to generate these estimates using a convenient nomogram enables informed clinical decision-making. Compared to the currently used tumor staging systems, the nomogram showed higher prediction accuracy and prognostic value [22–24]. And we have used an unusually large dataset (43,330 patients from SEER) to establish and evaluate accurate nomograms for predicting survival in patients with classical Hodgkin lymphoma [25]. The greatest advantage of using the nomogram is that it does not divide patients into groups for prognosis, but facilitates the assessment of the prognosis of each patient.

MM is a very heterogeneous disease. Like other lymphoproliferative diseases, such as lymphoma, the stratified approach for MM is appropriate to ensure that patients are treated with optimal efficacies and reduced toxicities.

In the present study, the nomogram was developed based on 214 retrospective cases in China. The nomogram was used to predict the 1-, 2-, and 3-year OS of patients with newly diagnosed MM, based on five independent risk factors: age at diagnosis, clonal BM plasma cells, serum LDH, serum *β*2-microglobulin, and del (17p).

Age at diagnosis has been demonstrated to be a significant prognostic factor in previous studies. A large study with a sample of 10549 patients with MM showed that, among patients in North America, Europe, and Japan who were 50−80 years old, when age was increased by 10 years, the median progression-free survival (PFS) was reduced by 0.7−1.0 years [26]. Ludwig et al. found that, regardless of traditional or high-dose chemotherapy, the median OS of patients with MM who were younger than 50 years was significantly longer than that of corresponding patients who were older than 50 years [27]. As seen in the nomograms that we have presented, the age at diagnosis had a strong prognostic association with OS.

The different clonal BM plasma cells are main diagnostic criteria. It has been suggested that the number of clonal BM plasma cells can be used as an indicator of prognosis [5]. The increase in expression levels of serum *β*2-microglobulin, the human white blood cell antigen-II on the surface of the B-lymphocytes, is related to the high tumor burden and kidney damage in patients with MM. The highest relative risk of chromosomal abnormalities in B lymphocytes and plasma cell tumors have independent prognostic significance. In 2005, serum *β*2-microglobulin combined serum protein level was proved to be the simplest staging index for MM, and the ISS was established [5]. In this study, serum *β*2-microglobulin was an independent risk factor for OS. High levels of serum LDH suggest high tumor burden and proliferation of MM. In recent studies, it has been seen that patients with high serum levels of LDH are likely to show the development of extramedullary plasmacytomas [28]. It has been demonstrated that combining LDH levels and FISH along with the ISS stage could significantly improve the prognostic assessment of PFS and OS, according to the R-ISS [6].

In recent years, with the progress in and application of cytogenetics and molecular biology, it has been found that most patients with MM have cytogenetic abnormalities. The identification of specific cytogenetic abnormalities by iFISH has become an important procedure for the prognostic stratification of MM. The most common abnormalities include del (17p), 1q21 gain, t (4;14), t (14;16), and t (11;14). Presently, the R-ISS, IMWG, and mSMART staging systems include the cytogenetic abnormalities in the prognosis of MM. Of these, the aberrations of t (4;14), t (14;16), and del (17p) are associated with adverse outcomes, whereas the translocation of t (11;14) is associated with relatively better outcomes. Sawyer et al. found that 1q21 gain occurred in most late stage MMs, in light of its involvement in the development and progression of MM. Patients with refractory or relapsed MMs with 1q21 gain have shorter survival times; this may also prove that 1q21 gain is an adverse prognostic factor [29, 30]. Deletion of 17p13 leads to the loss of heterozygosity of TP53 and is considered a high-risk feature in MM [17, 31]. Deletion of the p53 gene causes MM cells to lose the abilities of proliferation, differentiation, and apoptosis, eventually leading to the abnormal proliferation of tumor cells. Most staging systems for MM have classified del (17p) as a high-risk genetic abnormality. The PFS and OS could not be improved, even with high-dose chemotherapy plus stem cell transplantation [32]. The results of this study show that del (17p) was an independent risk factor for OS. However, in this study, the aberrations of 1q21 gain, t (4;14), t (14;16), and t (11;14) did not show statistically significant differences, probably because of the small sample size in this study. Further studies are needed to clarify this.

The nomogram showed good performance for predicting OS by the C-index (0.749 and 0.711 for the training and validation cohorts, respectively) and the calibration curves. Compared with the other five currently used staging systems, the nomogram showed higher predictive accuracy for survival.

This study had several limitations. First, this was a retrospective study; therefore, it was subject to inherent and unavoidable biases. Second, the nomogram was established based on data obtained from a single institution in China; hence, it is necessary to expand the sample size and validate the results by comparing them with those from other centers. Third, although the C-index of the nomogram was good, it was not excellent. Many other factors may influence the prognosis [1, 33], and further studies are needed to improve the nomogram. Despite these limitations, the present study is the first to apply the nomogram model to predict the survival of patients with MM.

## 5. Conclusions

The nomogram, as proposed in this study, can objectively and accurately predict the prognosis of patients with newly diagnosed MM. Further studies are needed to determine whether it can be applied to other patient groups.

## Figures and Tables

**Table 1:** Clinical and laboratory characteristics of patients with newly diagnosed multiple myeloma.

Characteristics	Training cohort		Validation cohort	
	(n = 214)		(n = 90)	
	Number	%	Number	%
Age at diagnosis, years				
Median ± SD	60.2 ± 10.6		60.9 ± 11.1	
Range	34−89		34−84	
Sex				
Male	121	56.5	51	56.7
Female	93	43.5	39	43.3
Clonal BM plasma cells, %				
Median	36.4 ± 24.8		38.2 ± 26.8	
Range	0.5−97.0		0.5−97.0	
Hemoglobin				
≥100 g/L	91	42.5	41	45.6
<100 g/L	123	57.5	49	54.4
Serum albumin				
≥35 g/L	73	34.1	29	32.2
<35 g/L	141	65.9	61	67.8
Serum LDH				
<250 U/L	178	83.2	74	82.2
≥250 U/L	36	16.8	16	17.8
Serum creatinine				
<177 *μ*mol/L	172	80.4	72	80.0
≥177 *μ*mol/L	42	19.6	18	20.0
Serum calcium				
<2.75 mmol/L	200	93.5	83	92.2
≥2.75 mmol/L	14	6.5	7	7.8
Serum *β*2-microglobulin				
<5.5 mg/L	120	56.1	48	53.3
≥5.5 mg/L	94	43.9	42	46.7
1q21 gain				
Negative	119	55.6	54	60.0
Positive	95	44.4	36	40.0
del (17p)				
Negative	186	86.9	80	88.9
Positive	28	13.1	10	11.1
t (4;14)				
Negative	189	88.3	83	92.2
Positive	25	11.7	7	7.8
t (11;14)				
Negative	169	79.0	69	76.7
Positive	45	21	21	23.3
t (14;16)				
Negative	206	96.3	85	94.4
Positive	8	3.7	5	5.6

Abbreviations: SD, standard deviation; BM, bone marrow; LDH, lactate dehydrogenase.

**Table 2:** Multivariate analysis of the overall survivals of patients in the training cohort.

Variables	Overall survival		
	HR	95% CI	*P*
Age at diagnosis, years	1.025	1.001−1.049	0.042
Clonal BM plasma cells	1.026	1.016−1.035	< 0.001
Serum albumin			
*⩾*35 g/L			
<35 g/L	1.043	0.589−1.846	0.885
Serum LDH			
<250 U/L			
*⩾*250 U/L	1.769	1.002−3.124	0.049
Serum *β*2-microglobulin			
<5.5 mmol/L			
≥5.5 mmol/L	1.709	1.041−2.807	0.034
1q21 gain (positive vs negative)	1.091	0.660−1.803	0.735
del (17p) (positive vs negative)	3.008	1.654−5.468	< 0.001

Abbreviations: HR, hazard ratio; CI, confidence interval; BM, bone marrow; LDH, lactate dehydrogenase.

**Table 3:** The C-indices for the nomogram and the five currently used staging systems for multiple myeloma to predict overall survival in patients.

Staging systems	Training cohort		Validation cohort	
	C-index	95% CI	C-index	95% CI
Nomogram	0.749	0.693−0.805	0.711	0.650−0.772
ISS	0.576	0.515−0.637	0.637	0.553−0.720
R-ISS	0.625	0.567−0.683	0.651	0.566−0.736
D-S	0.567	0.510−0.624	0.529	0.446−0.612
mSMART	0.583	0.524−0.642	0.620	0.532−0.708
IMWG	0.513	0.453−0.573	0.578	0.484−0.672

Abbreviations: C-index, index of concordance; CI, confidence interval; ISS, International Staging System; R-ISS, Revised-International Staging System; D-S, Durie-Salmon Staging System; mSMART, Mayo Stratification of Myeloma and Risk-Adapted Therapy; IMWG, International Myeloma Working Group risk stratification.

## Data Availability

The data used to support the findings of this study are available from the corresponding author upon request.

## Abbreviations

ASCT: Autologous stem cell transplantation
AUC: Area under the curve
BM: Bone marrow
CI: Confidence interval
C-index: Index of concordance
D-S: Durie-Salmon staging system
HR: Hazard ratio
iFISH: Interphase fluorescence in situ hybridization
IMWG: International Myeloma Working Group
ISS: International Staging System
LDH: Lactate dehydrogenase
MM: Multiple myeloma
mSMART: Mayo Stratification of Myeloma and Risk-Adapted Therapy
OS: Overall survival
PFS: Progression-free survival
R-ISS: Revised-International Staging System
TP53: Tumor protein p53.
